# The Efficacy and Safety of Canagliflozin by Frailty Status in Participants of the CANVAS and CREDENCE Trials

**DOI:** 10.1111/jgs.19444

**Published:** 2025-03-19

**Authors:** Tu N. Nguyen, Jie Yu, Vlado Perkovic, Meg Jardine, Kenneth W. Mahaffey, Clara K. Chow, Clare Arnott, Richard I. Lindley

**Affiliations:** ^1^ Westmead Applied Research Centre, Sydney Medical School, Faculty of Medicine and Health University of Sydney Sydney New South Wales Australia; ^2^ The George Institute for Global Health University of New South Wales Sydney New South Wales Australia; ^3^ Cardiovascular Department John Hunter Hospital Newcastle New South Wales Australia; ^4^ Hunter Medical Research Institute University of Newcastle Newcastle New South Wales Australia; ^5^ NHMRC Clinical Trials Centre, Faculty of Medicine and Health University of Sydney Sydney New South Wales Australia; ^6^ Concord Repatriation General Hospital Sydney New South Wales Australia; ^7^ Stanford Center for Clinical Research Stanford University School of Medicine Stanford California USA; ^8^ Department of Cardiology Westmead Hospital Sydney New South Wales Australia; ^9^ Department of Cardiology Royal Prince Alfred Hospital Sydney Australia; ^10^ Sydney Medical School University of Sydney Sydney Australia

**Keywords:** adverse outcomes, canagliflozin, CANVAS trial, CREDENCE trial, diabetes, frailty, safety

## Abstract

**Background:**

Sodium‐glucose cotransporter 2 (SGLT2) inhibitors have been shown to improve renal and cardiovascular outcomes in patients with type 2 diabetes. Limited evidence exists about the efficacy and safety of SGLT2 inhibitors in patients with frailty.

**Methods:**

This was a post hoc pooled, participant‐level data analysis of the CANVAS Program (CANVAS and CANVAS‐R) and the CREDENCE trial. We examined the effect of canagliflozin on: (1) Major adverse cardiovascular events (MACE), (2) Cardiovascular mortality, (3) all‐cause mortality, and (4) key safety outcomes. Frailty was defined by a Frailty Index (FI) based on a deficit accumulation approach (FI > 0.25: frail). Cox proportional‐hazard models were used to estimate the efficacy and safety of canagliflozin overall and according to frailty status.

**Results:**

There were 14,543 participants (10,142 from the CANVAS Program, 4401 from the CREDENCE trial). Their mean age was 63.2 years; 35.3% were female. Frailty was present in 56% of the study participants. The benefits of canagliflozin were observed in both the frail and non‐frail subgroups: HRs for MACE 0.80 (95% CI 0.70–0.90) in the frail versus 0.91 (95% CI 0.75–1.09) in the non‐frail (*p* for interaction = 0.27); HRs for cardiovascular mortality 0.79 (95% CI 0.67–0.95) in the frail versus 0.94 (95% CI 0.70–1.27) in the non‐frail (*p* for interaction = 0.38); HRs for all‐cause mortality 0.81 (95% CI 0.70–0.94) in the frail versus 0.93 (95% CI 0.74–1.16) in the non‐frail (*p* for interaction = 0.39). Adverse events were similar among frail and non‐frail participants, except for osmotic diuresis (HRs 1.67, 95% CI 1.22–2.28 in the frail vs. 3.05, 95% CI 2.13–4.35 in the non‐frail, p for interaction = 0.01).

**Conclusions:**

Canagliflozin improved cardiovascular and mortality endpoints in participants with type 2 diabetes irrespective of frailty status, with a similar safety profile. Our findings, in addition to those from other recent studies, provide evidence to support the introduction of SGLT2 inhibitor therapy in patients perceived to be frail.

**Trial Registration:** ClinicalTrials.gov CANVAS: NCT01032629; CANVAS‐R: NCT01989754; CREDENCE: NCT02065791

**Summary**

- Key points:
  ○ We found that the benefits of canagliflozin in participants with diabetes were consistent in both the frail and the non‐frail, with no evidence of an increase in adverse events in those defined as frail.
  ○ Our data suggest that there is likely to be important benefits if SGLT2 inhibitors are more widely used in frail patients with diabetes.
  ○ Our findings, in addition to those from other recent studies, provide evidence to support the introduction of SGLT2 inhibitors therapy in patients perceived to be frail.
- Why Does This Paper Matter?
  ○ SGLT2 inhibitors have significantly advanced the treatment paradigm for people with type 2 diabetes, chronic kidney disease, and heart failure.
  ○ However, there is still limited evidence regarding their efficacy and safety in vulnerable populations, particularly among those who are frail.
  ○ Our findings provide evidence to support the introduction of SGLT2 inhibitors therapy in patients perceived to be frail.
  ○ Ongoing research is imperative to fully understand the safety of SGLT2 inhibitors in older individuals with frailty, ensuring that all patients, including those with frailty, can safely and effectively benefit from the therapeutic potential of this medication.
  ○ Considering the high prevalence of frailty in patients with type 2 diabetes and cardiovascular disease, we recommend that frailty be prospectively measured at baseline in all future cardio‐metabolic trials involving older adults, and that this baseline variable should become as routine as age and sex.

## 1 Introduction

Sodium–glucose cotransporter 2 (SGLT2) inhibitors have been shown to improve clinical and mortality outcomes in individuals with type 2 diabetes, chronic kidney disease, and heart failure [1, 2]. SGLT2 inhibitors are a class of medication that prevent the reabsorption of glucose and sodium in the renal proximal convoluted tubule. SGLT2 inhibitors have been used in clinical practice for 10 years, with canagliflozin being the first SGLT2 inhibitor approved by the FDA for the treatment of type 2 diabetes in 2013, followed by dapagliflozin and empagliflozin in 2014, and sotagliflozin and bexagiflozin in 2023. Recent meta‐analyses suggested cardiovascular and renal benefits of SGLT2 inhibitors are consistent irrespective of age [3, 4, 5]. There is limited evidence, however, on the benefits and safety of SGLT2 inhibitors for those who are frail.

Frailty, defined as a state of vulnerability that carries an increased risk of poor outcomes in older adults, is common [6]. The topic of frailty and its implications for clinical practice becomes increasingly important as populations worldwide are living longer [7]. The aging process is heterogeneous, with individuals aging at different rates and in various ways [6]. As people age, their chronological age—simply the number of years they have lived—does not always provide an accurate reflection of their biological age [8]. Capturing the heterogeneity of aging through frailty enables better alignment of clinical decisions with patient risk profiles, challenging the traditional reliance on chronological age. The tendency in medical practice has often been to base treatment decisions more heavily on chronological age, which can lead to suboptimal patient care for older adults and prevent them from accessing the potential benefits of novel therapies designed to improve life expectancy and quality of life. Several studies have shown that responses to therapies are altered in frail people, including reduced immune responses to influenza and pneumococcal vaccines [9, 10, 11], and reduced responses to antithrombotic medications [12, 13]. The identification of frailty as a part of routine clinical assessments and wider population screening in older people has been recommended as it may help identify a subgroup at higher risk of adverse effects in whom treatment adjustment should be considered [14].

Clinicians may be hesitant to prescribe SGLT2 inhibitors for frail older patients due to uncertainty of the risk–benefit profile for this population. Recent evidence from the DELIVER (Dapagliflozin Evaluation to Improve the Lives of Patients With Preserved Ejection Fraction Heart Failure) [15] and DAPA‐HF (Dapagliflozin and Prevention of Adverse Outcomes in Heart Failure) [16] trials in heart failure populations suggests that patients with frailty may obtain greater absolute benefits with dapagliflozin. Canagliflozin has been shown to reduce cardiovascular events in patients with type 2 diabetes and elevated risk of cardiovascular disease in the CANVAS (Canagliflozin Cardiovascular Assessment Study) Program [17], and to reduce the risk of kidney failure and cardiovascular events in patients with type 2 diabetes and kidney disease in the CREDENCE (Canagliflozin and Renal Events in Diabetes with Established Nephropathy Clinical Evaluation) trial [18]. In this study, we aimed to investigate the impact of frailty on the efficacy and safety of canagliflozin therapy. Our specific objectives were to: (1) develop a bespoke trial Frailty Index in participants with type 2 diabetes in the CANVAS Program and CREDENCE trial; (2) examine the relationship of this trial Frailty Index on subsequent adverse outcomes among participants; and (3) examine the impact of frailty on the efficacy and safety of canagliflozin among participants.

## 2 Methods

This was a post hoc individual patient‐level analysis of the combined data of the CANVAS Program and CREDENCE trial. The CANVAS Program and CREDENCE trial received ethics approval from the ethics committee of each study center. All participants provided written informed consent.

In brief, the CANVAS Program integrated data from two double‐blind, randomized controlled trials (CANVAS and CANVAS‐R) involving a total of 10,142 participants with type 2 diabetes and high cardiovascular risk from 667 sites in 30 countries. Participants in each trial were randomly assigned to receive canagliflozin (300 or 100 mg) or placebo and were followed for a mean of 3.62 years [17]. Participants were men and women with type 2 diabetes and were either 30 years of age or older with a history of symptomatic atherosclerotic cardiovascular disease or aged ≥ 50 years with two or more risk factors for cardiovascular disease duration of diabetes of at least 10 years, systolic blood pressure > 140 mmHg while they were receiving one or more antihypertensive agents, current smoking, microalbuminuria or macroalbuminuria, or high‐density lipoprotein (HDL) cholesterol level of less than 1.0 mmol/L. Participants were required to have an estimated glomerular filtration rate (eGFR) at entry of more than 30 mL per minute per 1.73 m^2^ of body‐surface area and to meet a range of other criteria.

The CREDENCE trial was a double‐blind, randomized trial involving 4401 participants with type 2 diabetes and albuminuric chronic kidney disease from 690 sites in 34 countries [18]. Participants were randomized to receive canagliflozin at a dose of 100 mg daily or placebo and were followed for a mean duration of 2.62 years. All the participants had an eGFR of 30 to < 90 mL per minute per 1.73 m [2] of body surface area and albuminuria, and were treated with renin–angiotensin system blockade.

In the CANVAS Program, the primary outcome was major adverse cardiovascular events (MACE): the composite of cardiovascular (CV) mortality, non‐fatal myocardial infarction, and non‐fatal stroke. In the CREDENCE trial, the primary outcome was a composite of end‐stage kidney disease (dialysis for at least 30 days, kidney transplantation, or an estimated GFR of < 15 mL per minute per 1.73 m^2^ sustained for at least 30 days according to central laboratory assessment), doubling of the serum creatinine level from baseline (average of randomization and pre‐randomization value) sustained for at least 30 days according to central laboratory assessment, or death from renal or cardiovascular disease.

### 2.1 Frailty Index

In this study, we used the baseline data from the CANVAS Program and CREDENCE trial to create a Frailty Index measure based on a deficit accumulation approach described by Rockwood and coauthors [19], referencing recent studies based on clinical trial data [15, 16, 20].

A total of 27 variables were identified as suitable to construct a Frailty Index (Table S1). These variables include cardiovascular risk factors and comorbidities (smoking, obesity, hypertension, heart failure, coronary heart disease, cerebrovascular disease, peripheral artery disease, chronic kidney disease, fracture, amputation, diabetic retinopathy, diabetic nephropathy, diabetic neuropathy), laboratory measures (serum HbA1c, Albumin/Creatinin ratio, high‐density lipoprotein cholesterol, low‐density lipoprotein cholesterol, total cholesterol, triglyceride, sodium, potassium, hemoglobin), physical measurements (systolic blood pressure, diastolic blood pressure, underweight based on body mass index), polypharmacy, and diabetes duration. All variables included in the Frailty Index were scored such that 0 signified the absence of a factor, while the presence of a deficit was given a score of 1. The Frailty Index (FI) was constructed for an individual by summing the scores of all variables and dividing by the total number of variables considered. The Frailty Index values range from 0 to 1, and the cut‐point to identify frailty was FI > 0.25 as applied in previous studies [21, 22].

### 2.2 Outcome Variables

In this study, our primary outcomes overall and in subgroups defined by a FI > 0.25 were:

1. MACE: CV mortality, non‐fatal myocardial infarction, and non‐fatal stroke
2. CV mortality
3. All‐cause mortality

Safety outcomes assessed overall, and in subgroups defined by frailty, were total serious adverse events, hypoglycemia, renal‐related adverse events, acute kidney injury, diabetic ketoacidosis, volume depletion, osmotic diuresis, hyperkalemia, fracture, amputation, urinary tract infections (UTI), and infection of female/male genitalia.

### 2.3 Statistical Analysis

Data were summarized, with continuous variables presented as means (standard deviation), and categorical variables as frequencies and percentages.

*To examine the impact of frailty on adverse outcomes*, two sets of models were fitted; first, we constructed Cox proportional‐hazard models with frailty as the only predictor variable. Second, we constructed models that included canagliflozin, age, and sex, in addition to frailty, as there is strong evidence of the impact of sex and age on frailty [23, 24].

To examine the effect of canagliflozin on the study outcomes in frail and non‐frail participants, crude incidence rates of outcomes by canagliflozin treatment were calculated. To examine the impact of frailty on the efficacy and safety of canagliflozin, we fitted Cox proportional‐hazard models including an interaction term between canagliflozin treatment and frailty status. Results were presented as hazard ratios (HRs) and 95% confidence intervals (CIs) for the frail and non‐frail. Interaction terms between frailty status and canagliflozin treatment were included to model the homogeneity of treatment effects in the frail and the non‐frail, and *p* values for interaction are presented to guide any statistically significant differences between the frail and non‐frail.

We conducted sensitivity analyses for participants aged 65 or above and for participants in each trial separately. We also conducted additional analyses with three levels of frailty (FI ≤ 0.25, FI from 0.25 to 0.35, and FI > 0.35).

All *p* values were two‐sided, and those less than 0.05 were considered to indicate statistical significance [25, 26]. Analysis of the data were performed using SPSS for Windows 27.0 (IBM Corp., Armonk, NY, USA) and SAS 9.4.

## 3 Results

There were 14,543 participants (10,142 from the CANVAS Program, 4401 from the CREDENCE trial). Their mean age was 63.2 years; 35.3% were female. The baseline characteristics of participants by frailty status are presented in Table 1.

**TABLE 1:** Baseline characteristics of the study participants overall and by frailty status.

	All (*n* = 14,543)	Frail (*n* = 8080)	Non‐frail (*n* = 6463)	*p*
Age, years (mean ± SD)	63.22 ± 8.55	63.19 ± 8.63	63.25 ± 8.44	0.117
< 65 years *n* (%)	7922 (54.5%)	4362 (54.0%)	3560 (55.1%)	0.187
≥ 65 years *n* (%)	6621 (45.5%)	3718 (46.0%)	2903 (44.9%)	
Sex, *n* (%)				
Female	5127 (35.3%)	2905 (36.0%)	2222 (34.4%)	0.049
Male	9416 (64.7%)	5175 (64.0%)	4241 (65.6%)	
Trials				
CANVAS	10,142 (69.7%)	4381 (54.2%)	5761 (89.1%)	< 0.001
CREDENCE	4401 (30.3%)	3699 (45.8%)	702 (10.9%)	
Region				
Central/South America	1962 (13.5%)	1196 (14.8%)	766 (11.9%)	< 0.001
Europe	4473 (30.8%)	2169 (26.8%)	2304 (35.6%)	
North America	3612 (24.8%)	1996 (24.7%)	1616 (25.0%)	
Rest of the world	4496 (30.9%)	2719 (33.7%)	1777 (27.5%)	
Race				
American Indian or Alaska Native	116 (0.8%)	79 (1.0%)	37 (0.6%)	< 0.001
Asian	2161 (14.9%)	992 (12.3%)	1169 (18.1%)	
Black or African American	560 (3.9%)	352 (4.4%)	208 (3.2%)	
Multiple	114 (0.8%)	63 (0.8%)	51 (0.8%)	
Native Hawaiian or other Pacific Islander	48 (0.3%)	33 (0.4%)	15 (0.2%)	
Other	649 (4.5%)	305 (3.8%)	344 (5.3%)	
Unknown	20 (0.2%)	16 (0.2%)	4 (0.1%)	
White	10,875 (74.8%)	6240 (77.2%)	4635 (71.7%)	
Diabetes duration, years (mean ± SD)	14.23 ± 8.10	15.60 ± 8.59	12.50 ± 7.07	< 0.001
Baseline HbA1c, %	8.25 ± 1.06	8.42 ± 1.15	8.05 ± 0.91	< 0.001
Baseline eGFR (mL/min)	70.33 ± 21.94	63.92 ± 21.41	78.34 ± 19.85	< 0.001
Baseline urine albumin‐to‐creatinine ratio				
< 30	7038 (48.8%)	2581 (32.2%)	4457 (69.6%)	< 0.001
30–300	2762 (19.1%)	1479 (18.4%)	1283 (20.0%)	
301–3000	4074 (28.2%)	3455 (43.0%)	619 (9.7%)	
> 3000	560 (3.9%)	512 (6.4%)	48 (0.7%)	
Baseline systolic blood pressure (mmHg)	137.65 ± 15.79	139.58 ± 16.09	135.24 ± 15.07	< 0.001
Baseline diastolic blood pressure (mmHg)	77.89 ± 9.58	78.52 ± 10.03	77.09 ± 8.92	< 0.001
Baseline total cholesterol (mmol/L)	4.45 ± 1.21	4.71 ± 1.34	4.13 ± 0.93	< 0.001
Baseline LDL cholesterol (mmol/L)	2.35 ± 0.98	2.52 ± 1.09	2.15 ± 0.78	< 0.001
Baseline HDL cholesterol (mmol/L)	1.17 ± 0.33	1.13 ± 0.34	1.21 ± 0.30	< 0.001
Baseline triglycerides (mmol/L)	2.09 ± 1.49	2.39 ± 1.75	1.71 ± 0.94	< 0.001
Smokers, *n* (%)	2445 (16.8%)	1500 (18.6%)	945 (14.6%)	< 0.001
BMI group, *n* (%)				
Underweight (BMI < 18.50)	25 (0.2%)	15 (0.2%)	10 (0.2%)	< 0.001
Normal weight (BMI 18.50–24.99)	1500 (10.3%)	650 (8.1%)	850 (13.2%)	
Overweight (BMI (25.00–29.99)	4608 (31.8%)	2069 (25.7%)	2539 (39.4%)	
Obese (BMI ≥ 30.00)	8375 (57.7%)	5329 (66.1%)	3046 (47.3%)	
Comorbidities				
History of hypertension	13,385 (92.0%)	7820 (96.8%)	5565 (86.1%)	< 0.001
History of heart failure	2113 (14.5%)	1822 (22.5%)	291 (4.5%)	< 0.001
History of coronary heart disease	7034 (48.4%)	4202 (52.0%)	2832 (43.8%)	< 0.001
History of cerebrovascular disease	2658 (18.3%)	1960 (24.3%)	698 (10.8%)	< 0.001
History of peripheral artery disease	3159 (21.7%)	2512 (31.1%)	647 (10.0%)	< 0.001
History of fracture	2925 (20.1%)	1949 (24.1%)	976 (15.1%)	< 0.001
History of amputation	472 (3.2%)	436 (5.4%)	36 (0.6%)	< 0.001
History of diabetic retinopathy	4011 (27.6%)	3355 (41.5%)	656 (10.2%)	< 0.001
History of diabetic nephropathy	6175 (42.5%)	5016 (62.1%)	1159 (17.9%)	< 0.001
History of diabetic neuropathy	5257 (36.1%)	4197 (51.9%)	1060 (16.4%)	< 0.001
Chronic kidney disease	4663 (32.1%)	3765 (46.6%)	898 (13.9%)	< 0.001
Polypharmacy (using ≥ 5 medications)	10,684 (73.5%)	6462 (80.0%)	4222 (65.3%)	< 0.001
Randomized treatments with canagliflozin *n* (%)	7997 (55.0%)	4344 (53.8%)	3653 (56.5%)	< 0.001

*Note*: Continuous data are presented as mean (standard deviation); categorical data are shown as *n* (%).

Abbreviations: BMI, body mass index; CABG, coronary artery bypass grafting; ECG Electrocardiogram; MMSE, mini‐mental state examination; TIA, transient ischemic attack.

The Frailty Index values were approximately normally distributed, with a mean of 0.264 and a standard deviation of 0.103 (ranging from 0 to 0.667, median of 0.259) (Figure S1). Using the cut‐point of 0.25, the prevalence of frailty was 56% (8080/14543) in the study participants (55.0% in men and 57% in women, *p* = 0.049).

### 3.1 The Relationship of Baseline Frailty to Subsequent Adverse Outcomes

The unadjusted rates for outcomes in the study participants by frailty status are presented in Table 2. Frail participants had a higher incidence of the MACE, all‐cause mortality, and CV mortality. In Cox models adjusted for canagliflozin, age, and sex, frailty was independently associated with increased adverse outcomes (Table 2). The Kaplan–Meier curves for frail and non‐frail participants are presented in Figure S2.

**TABLE 2:** The impact of frailty on the study outcomes in all participants.

Outcomes	Unadjusted HR (95% CI)	Adjusted HR (95% CI)
Composite event (cardiovascular mortality/myocardial infarction/stroke)	2.10 (1.89–2.35)	2.09 (1.87–2.34)
All‐cause mortality	2.17 (1.91–2.48)	2.15 (1.88–2.45)
Cardiovascular mortality	2.80 (2.37–3.32)	2.78 (2.34–3.29)

*Note*: Adjusted for age, sex and canagliflozin treatment.

### 3.2 The Effect of Canagliflozin on Clinical Outcomes by Frailty Status

**FIGURE 1:**
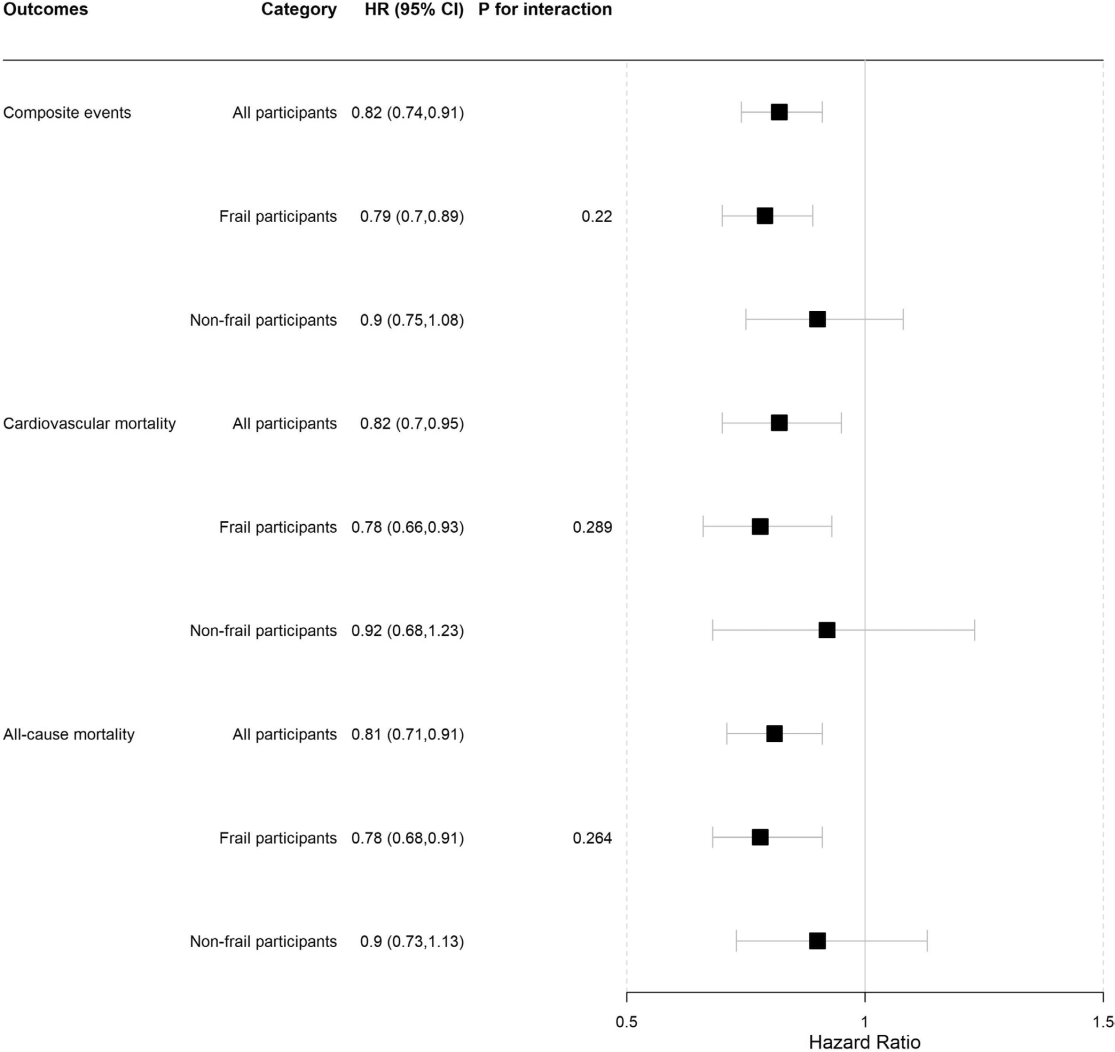
Unadjusted hazard ratios of canagliflozin treatment versus placebo on the study outcomes in frail and non‐frail participants.

The benefits of canagliflozin were seen in both the frail and non‐frail participants with no heterogeneity by frailty identified for any outcome. For MACE: HR 0.80 (95% CI 0.70–0.90) in frail participants versus HR 0.91 (95% CI 0.75–1.09) in the non‐frail (*p* for interaction = 0.27). For CV mortality: HR 0.79 (95% CI 0.67–0.95) in frail participants versus HR 0.94 95% CI (0.70–1.27) in the non‐frail (*p* for interaction = 0.38). For all‐cause mortality: HR 0.81 (95% CI 0.70–0.94) in frail participants versus HR 0.93 (95% CI 0.74–1.16) in the non‐frail (*p* for interaction = 0.39) (Figure 1).

### 3.3 The Effect of Canagliflozin on Safety Outcomes by Frailty Status

Osmotic diuresis was less common in frail participants compared to the non‐frail (HRs 1.67, 95% CI 1.22–2.28 in the frail vs. 3.05, 95% CI 2.13–4.35 in the non‐frail, *p* for interaction = 0.01). Other adverse events were similar among frail and non‐frail participants, including hypoglycemia (HRs 1.08, 95% CI 0.92–1.26 in the frail vs. 0.94, 95% CI 0.77–1.16 in the non‐frail, *p* for interaction = 0.37), volume depletion (HRs 1.32, 95% CI 1.07–1.62 in the frail vs. 1.20, 95% CI 0.89–1.61 in the non‐frail, *p* for interaction = 0.59), renal related adverse events (HRs 0.78, 95% CI 0.68–0.90 in the frail vs. 0.89, 95% CI 0.65–1.22 in the non‐frail, *p* for interaction = 0.41), acute kidney injury (HRs 0.80, 95% CI 0.62–1.05 in the frail vs. 0.67, 95% CI 0.36–1.24 in the non‐frail, *p* for interaction = 0.72), hyperkalemia (HRs 0.84, 95% CI 0.68–1.04 in the frail vs. 0.94, 95% CI 0.58–1.54 in the non‐frail, *p* for interaction = 0.79), diabetic ketoacidosis (HRs 7.33, 95% CI 1.69–31.85 in the frail vs. 1.78, 95% CI 0.47–6.83 in the non‐frail, *p* for interaction = 0.19), fracture (HRs 1.08, 95% CI 0.86–1.34 in the frail vs. 1.33, 95% CI 1.04–1.71 in the non‐frail, *p* for interaction = 0.13), amputation (HRs 1.41, 95% CI 1.10–1.82 in the frail vs. 1.96, 95% CI 1.04–3.71 in the non‐frail, *p* for interaction = 0.29), urinary tract infection (HRs 1.001, 95% CI 0.85–1.18 in the frail vs. 1.26, 95% CI 1.02–1.56 in the non‐frail, *p* for interaction = 0.07), female genital mycotic infection (HRs 4.22, 95% CI 2.55–6.97 in the frail vs. 3.72, 95% CI 2.29–6.05 in the non‐frail, *p* for interaction = 0.76), male genital mycotic infection (HRs 4.69, 95% CI 3.04–7.21 in the frail vs. 3.67, 95% CI 2.70–5.00 in the non‐frail, *p* for interaction = 0.29). (Table 3).

**TABLE 3:** Cox proportional hazards models of canagliflozin on the safety outcomes in frail and non‐frail participants.

Outcomes	All participants	Frail	Non‐frail	P for interaction between frailty and canagliflozin intervention
	Unadjusted HRs (95% CI)	Unadjusted HRs (95% CI)	Unadjusted HRs (95% CI)	
Hypoglycemia	1.03 (0.91–1.17)	1.08 (0.92–1.26)	0.94 (0.77–1.16)	0.37
Urinary tract infection	1.09 (0.96–1.24)	1.001 (0.85–1.18)	1.26 (1.02–1.56)	0.07
Renal related adverse events	0.81 (0.72–0.92)	0.78 (0.68–0.90)	0.89 (0.65–1.22)	0.41
Fracture	1.19 (1.01–1.40)	1.08 (0.86–1.34)	1.33 (1.04–1.71)	0.13
Volume depletion	1.29 (1.09–1.54)	1.32 (1.07–1.62)	1.20 (0.89–1.61)	0.59
Osmotic diuresis	2.22 (1.76–2.80)	1.67 (1.22–2.28)	3.05 (2.13–4.35)	0.01
Hyperkalemia	0.86 (0.71–1.05)	0.84 (0.68–1.04)	0.94 (0.58–1.54)	0.79
Amputation	1.50 (1.19–1.89)	1.41 (1.10–1.82)	1.96 (1.04–3.71)	0.29
Acute kidney injury	0.79 (0.62–1.004)	0.80 (0.62–1.05)	0.67 (0.36–1.24)	0.72
Diabetic ketoacidosis	3.94 (1.50–10.36)	7.33 (1.69–31.85)	1.78 (0.47–6.83)	0.19
Female genital mycotic infection	3.92 (2.76–5.56)	4.22 (2.55–6.97)	3.72 (2.29–6.05)	0.76
Male genital mycotic infection	3.97 (3.09–5.10)	4.69 (3.04–7.21)	3.67 (2.70–5.00)	0.29

Sensitivity analyses indicated that the study findings are consistent for participants aged 65 or above, for participants in each trial separately, and across the three levels of frailty (FI ≤ 0.25, FI from 0.25 to 0.35, and FI > 0.35) (Tables S3–S8).

## 4 Discussion

In this pooled post hoc analysis of the CANVAS Program and CREDENCE trial using individual patient‐level data, we were able to create a bespoke Frailty Index derived from the trial baseline variables. We found that the benefits of canagliflozin in participants with diabetes was consistent in both the frail and the non‐frail, with no evidence of an increase in adverse events in those defined as frail. While we identified greater absolute benefits for the frailer patients, there was no statistical interaction for frailty on these treatment end‐points. Importantly, although the frailer patients also had an increased risk of adverse events (including hypoglycemia, renal related adverse events, acute kidney injury, volume depletion, hyperkalemia, amputation), canagliflozin did not further increase this risk, and surprisingly, the frail subgroup had a lower risk of osmotic diuresis on treatment.

Importantly, our findings are consistent with the results from the DAPA‐HF and the DELIVER trials that included a patient population with heart failure with reduced ejection fraction (with and without type 2 diabetes). In the DELIVER trial (*n* = 6258), the benefit of dapagliflozin was similar across the range of frailty studied: the hazard ratio on the primary end point was 0.85 (95% CI, 0.68–1.06) in the non‐frail, 0.89 (0.74–1.08) in the more frail, and 0.74 (0.61–0.91) in the most frail (*p* value for interaction = 0.40) [15]. The improvement in health‐related quality of life with dapagliflozin occurred early and was greater in patients with a higher level of frailty [15]. Adverse reactions and treatment discontinuation were more common in participants with a greater degree of frailty. However, these events were not more frequent with dapagliflozin than with placebo irrespective of frailty class. In the DAPA‐HF, in patients with symptomatic heart failure with a left ventricular ejection fraction of 40% or less and elevated natriuretic peptide, analysis from 4742 participants showed that frailty was present in approximately 50% of the participants, and dapagliflozin improved all outcomes examined regardless of frailty status (with the absolute reductions being larger in more frail patients) [16]. While these analyses from the DELIVER and DAPA‐HF trials focused on the effect of frailty on responses to dapagliflozin in patients with heart failure, our analysis is the first to focus on the effect of frailty on responses to canagliflozin treatment in an exclusive type 2 diabetes population.

These results provide important new data for clinicians, with reassurance that this treatment is safe and effective, even in the presence of frailty. A significant number of hospitalized patients are identified as frail, making it crucial to ensure that our treatment options for such patients are based on reliable randomized controlled trial data. Our analysis showed that frail participants had similar adverse events, including any serious adverse events, hypoglycemia, renal‐related adverse events, acute kidney injury, volume depletion, hyperkalemia, and amputation. Interestingly, we found that osmotic diuresis rates were higher in the non‐frail compared to the frail, possibly due to better renal function in the non‐frail [27]. It may be attributable to the fact that the non‐frail group consists of participants with higher estimated glomerular filtration rates (eGFR is a component of the Frailty Index), which in turn suggests they have more functioning nephrons. Consequently, this results in a greater net effect, leading to increased diuresis. Frailty is characterized by the presence of multiple dysfunctions in various bodily systems, including the musculoskeletal, neuroendocrine, hematological, immune, and cardiovascular systems [6]. In frail individuals, the increased loss of muscle mass, reduced thirst, and decreased ability to concentrate urine are intrinsic characteristics that contribute to a chronic state of hypertonic dehydration [6, 27, 28]. Potential additional mechanisms of action of the SGLT2 inhibitors could help explain the augmented benefits we observed in the frail. Such mechanisms could include the capacity to evoke a metabolic and transcriptional state mimicking fasting characterized by calorie loss through urine, reduction in adipose tissue mass, and activation of catabolic pathways [29, 30]. SGLT2 inhibitors induce a state of starvation mimicry with notable effects including increased gluconeogenesis and ketogenesis, and the primary driver of gluconeogenesis and ketogenesis is the stimulation of SIRT1 (also known as sirtuin‐1 or silent information regulator 1). SIRT1 serves as a central regulator for numerous genes crucial to energy and cellular balance [30].

Our findings also supported the Rockwood cumulative deficits approach in defining frailty, as our trial Frailty Index identified those at increased vulnerability to adverse events. The prevalence of frailty in this study was broadly consistent with the reported frailty prevalence of around 50%–60% in other trials, such as the DAPA‐HF, DELIVER, PARADIGM‐HF (Prospective comparison of ARNI with ACE‐I to Determine Impact on Global Mortality and Morbidity in Heart Failure) and the ATMOSPHERE (Aliskiren Trial to Minimize Outcomes in Patients with Heart Failure) [15, 16, 31]. Frailty should be proactively assessed in all participants of clinical drug trials. However, developing a Frailty Index based on clinical trial data presents several challenges that should be carefully navigated to ensure accuracy and applicability. Clinical trial data often prioritize specific health outcomes related to the primary aims of the trial and may overlook or inadequately capture some indicators of frailty, such as cognitive decline, functional impairment, or social factors. Furthermore, the reliance on retrospective data poses a risk of bias and limits the ability to account for long‐term changes or trends in frailty progression. To ensure a Frailty Index is valid across different populations, several essential steps should be undertaken. There should be a comprehensive collection of data at baseline that includes a wide range of health indicators, from comorbidities to physical function, cognitive domains, socio‐economic factors, psychological factors, and quality of life. This will allow the index to account for the multifaceted nature of frailty, which can manifest differently across populations.

## 5 Strengths and Limitations

The strengths of this study reside in its rigorous analysis of individual participant data derived from randomized controlled trials and the internal consistency of the bespoke Frailty Index in identifying those at higher risk of poor outcomes. The study sample size was large and representative, with participants recruited from more than 30 countries in these randomized controlled trials. Our data suggest that there is likely to be important benefits if these drugs are more widely used in frail patients with diabetes.

One primary limitation of this study is the post hoc analysis design, which inherently carries a risk of bias. Additionally, the construction of the Frailty Index was hindered by the limited number of baseline variables available. If every participant in the study exhibits the same variable, the trial's Frailty Index may lose its ability to distinguish between varying levels of frailty. Consequently, our Frailty Index may not be directly comparable to others, as by necessity we were limited by available baseline trial data. Despite these limitations, our trial Frailty Index provided useful predictive information. Moreover, it is essential to acknowledge that trial populations generally tend to be healthier than the general population [32], meaning that the ‘frail’ individuals within the trial may not be as frail as those in the general population. Therefore, the study findings should be interpreted with caution, considering that the degree of frailty in the trial setting may not fully capture the reality faced by the frail adults in the general population.

## 6 Conclusions

We were able to retrospectively devise a Frailty Index that described an important group of patients with an increased risk of poor outcomes. Participants with frailty had similar relative benefits from canagliflozin treatments compared to non‐frail participants. Our findings, in addition to those from other recent studies, provide evidence to support the introduction of SGLT2 inhibitors therapy in patients perceived to be frail. Given the high prevalence of frailty in patients with type 2 diabetes and cardiovascular disease, we recommend that frailty be prospectively measured at baseline in all future cardio‐metabolic trials that include older people, and that this baseline variable becomes as routine as age and sex.

## Author Contributions

R.I.L. and T.N.N. conceived this study. T.N.N. and J.Y. conducted the statistical analyses. T.N.N. led the manuscript writing. All authors were involved in data interpretation. The manuscript was revised for important scientific content by all authors. All authors gave final approval of the version to be published. R.I.L. is the guarantor of this work.

## Disclosure

The CANVAS and CREDENCE trials were sponsored by Janssen Research & Development LLC. The sponsor however had no role in this analysis or manuscript.

## Conflicts of Interest

V.P. has received fees for Advisory Boards, Steering Committee roles, or Scientific Presentations from Abbvie, Astellas, AstraZeneca, Bayer, Baxter, BMS, Boehringer Ingelheim, Dimerix, Durect, Eli Lilly, Gilead, GSK, Janssen, Merck, Mitsubishi Tanabe, Mundipharma, Novartis, Novo Nordisk, Pfizer, Pharmalink, Relypsa, Retrophin, Sanofi, Servier, Vifor and Tricida. M.J. is supported by an Australian government NHMRC Investigator Grant; is responsible for research projects that have received funding from Amgen, Baxter, CSL, Dimerix, Eli Lilly, Gambro, Kensana and MSD; has received fees for Advisory, Steering Committee and/or Scientific Presentations from Akebia, Amgen, Astra Zeneca, Baxter, Bayer, Boehringer Ingelheim, Cesas Medical, Chinook/Novartis, CSL, Janssen, Medcon International, Medscape, MSD, NovoNordisk, Occurx, Roche and Vifor; with any consultancy, honoraria or travel support directed to her institution. K.W.M.'s financial disclosures can be viewed at http://med.stanford.edu/profiles/kenneth‐mahaffey. C.K.C. is supported by a National Health and Medical Research Council of Australia Investigator grant APP1195326. C.K.C. has received honoraria for speaker's fees and advisory work from Novo Nordisk, Novartis, Eli Lilly, Amgen. C.K.C. is a Board Member of the National Heart Foundation of Australia, and the Western Sydney Local Health District. C.A. has received honoraria or sat of Steering Committees/Advisory Boards for Astra Zeneca, Novo Nordisk and Amgen. R.I.L. declared that he was an event adjudicator for the CANVAS trials, for which he was reimbursed. T.N.N. and J.Y. declare no conflicts of interest.

## 7 Linked Articles

This publication is linked to a related editorial by Aliberti et al. To view this article, visit https://doi.org/10.1111/jgs.19443.

## Supporting information


**Figure S1.** The distribution of the Frailty Index in the participants.
**Figure S2.** The survival curves for frail and non‐frail participants.
**Table S1.** List of items for the construction of a Frailty Index based on the baseline data (*N* = 14,543).
**Table S2.** Incidence rates for outcomes in the study participants by frailty status.
**Table S3.** Cox proportional hazards models of canagliflozin on composite events in frail and non‐frail participants aged ≥ 65 (*N* = 6621).
**Table S4.** Hazard ratios (95% CI) for safety outcomes in participants aged ≥ 65 (*N* = 6621).
**Table S5.** Cox proportional hazards models of canagliflozin on composite events by frailty level.
**Table S6.** Cox proportional hazards models of canagliflozin on safety outcomes by frailty level in all participants (*N* = 14,543).
**Table S7.** Cox proportional hazards models of canagliflozin on composite events by frailty status in each trial separately.
**Table S8.** Cox proportional hazards models of canagliflozin on composite events by frailty level in each trial separately.
