# Supplementary material for: The Efficacy and Safety of Canagliflozin by Frailty Status in Participants of the CANVAS and CREDENCE Trials
Source: J Am Geriatr Soc. 2025 Mar 19;73(6):1787–96. doi: 10.1111/jgs.19444 (PMC12205298; doi:10.1111/jgs.19444)
Supplement: Supplementary file 1 — Figure S1. The distribution of the Frailty Index in the participants. Figure S2. The survival curves for frail and non‐frail participants. Table S1. List of items for the construction of a Frailty Index based on the baseline data (N = 14,543). Table S2. Incidence rates for outcomes in the study participants by frailty status. Table S3. Cox proportional hazards models of canagliflozin on composite events in frail and non‐frail participants aged ≥ 65 (N = 6621). Table S4. Hazard ratios (95% CI) for safety outcomes in participants aged ≥ 65 (N = 6621). Table S5. Cox proportional hazards models of canagliflozin on composite events by frailty level. Table S6. Cox proportional hazards models of canagliflozin on safety outcomes by frailty level in all participants (N = 14,543). Table S7. Cox proportional hazards models of canagliflozin on composite events by frailty status in each trial separately. Table S8. Cox proportional hazards models of canagliflozin on composite events by frailty level in each trial separately. [file JGS-73-1787-s001.pdf]

Supplementary data

Supplementary Figure 1. The distribution of the Frailty Index in the participants
Supplementary Figure 2. The survival curves for frail and non-frail participants
Supplementary Table 1. List of items for the construction of a Frailty Index based on the baseline data (N=14543)
Supplementary Table 2. Incidence rates for outcomes in the study participants by frailty status
Supplementary Table 3. Cox proportional hazards models of canagliflozin on composite events in frail and non-frail participants aged ≥ 65 (N=6621)
Supplementary Table 4. Hazard ratios (95% CI) for safety outcomes in participants aged ≥ 65 (N=6621)
Supplementary Table 5. Cox proportional hazards models of canagliflozin on composite events by frailty level
Supplementary Table 6. Cox proportional hazards models of canagliflozin on safety outcomes by frailty level in all participants (N=14543)
Supplementary Table 7. Cox proportional hazards models of canagliflozin on composite events by frailty status in each trial separately
Supplementary Table 8. Cox proportional hazards models of canagliflozin on composite events by frailty level in each trial separately

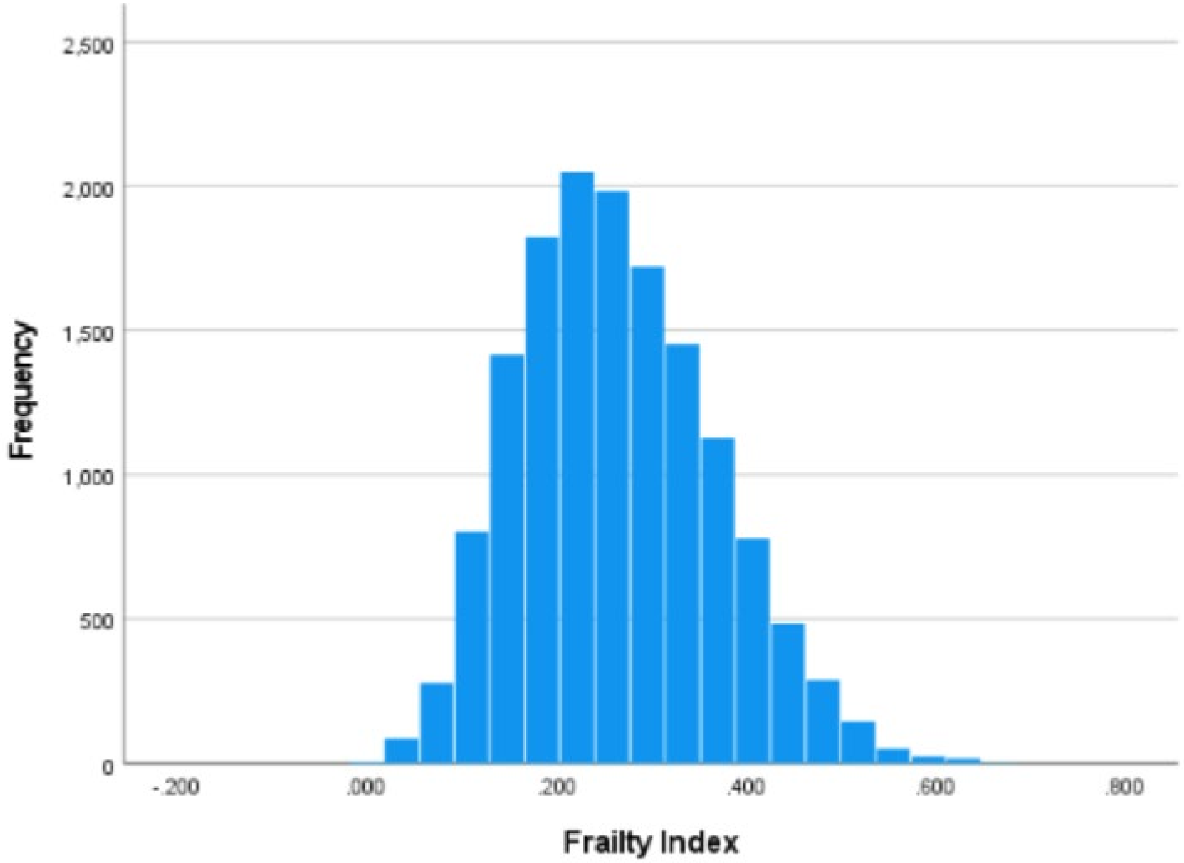

Supplementary Figure 1. The distribution of the Frailty Index in the participants

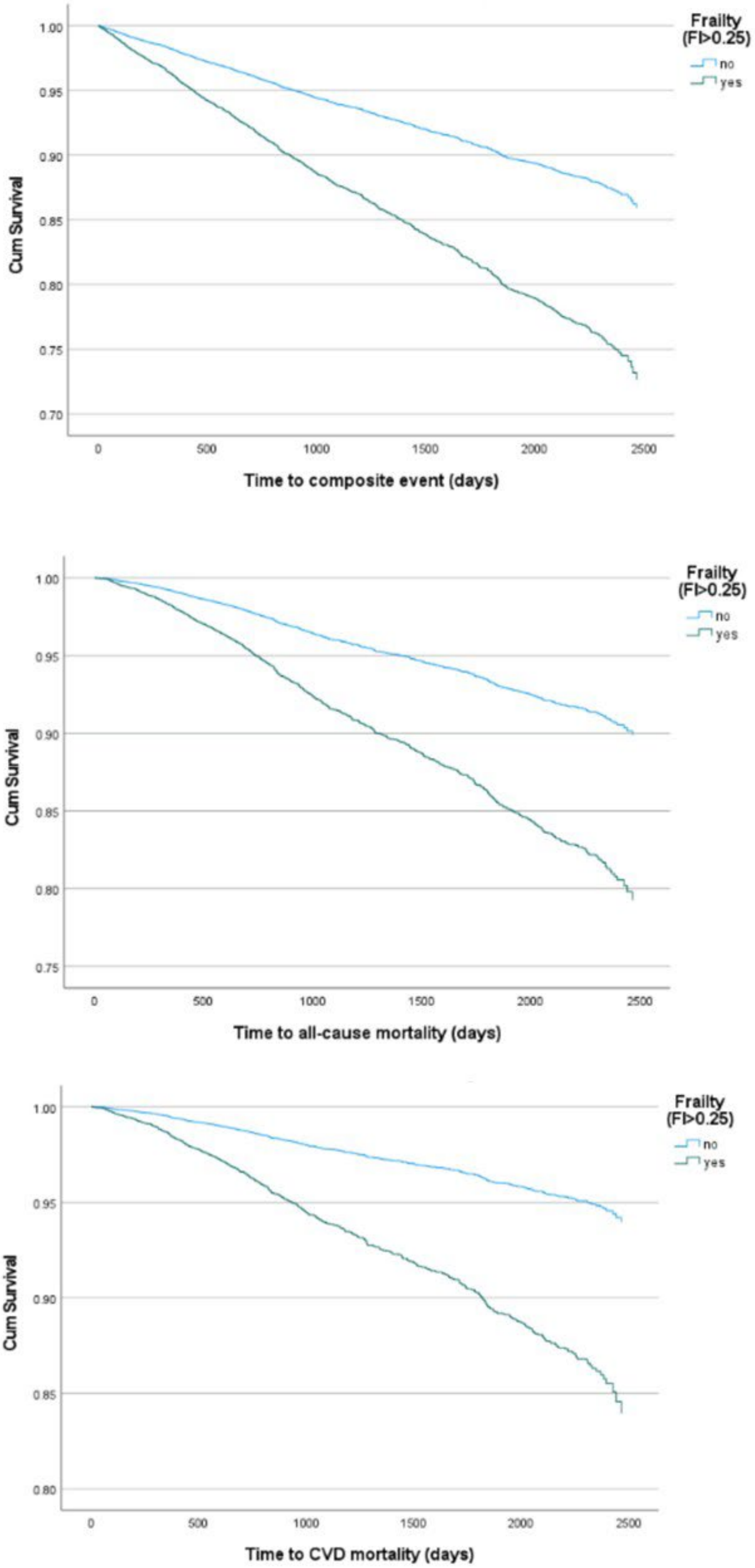

Supplementary Figure 2. The survival curves for frail and non-frail participants

Supplementary Table 1. List of items for the construction of a Frailty Index based on the baseline data (N=14543)

	Items	Scoring	Missing	Note
**Cardiovascular risk factors and comorbidities**				
1	Smoking	No: 0<br>Yes: 1	3	
2	Obesity	No: 0<br>Yes: 1	23 (0.2%)	Recode from BMI >=30.0
3	History of hypertension	No: 0<br>Yes: 1	0	
4	History of heart failure	No: 0<br>Yes: 1	0	
5	History of coronary heart disease	No: 0<br>Yes: 1	0	
6	History of cerebrovascular disease	No: 0<br>Yes: 1	0	
7	History of peripheral artery disease	No: 0<br>Yes: 1	0	
8	Chronic kidney disease	No: 0<br>Yes: 1	10 (0.1%)	Recode from GFR≥60 ml/min
9	History of fracture	No: 0<br>Yes: 1	0	
10	History of amputation	No: 0<br>Yes: 1	0	
11	History of diabetic retinopathy	No: 0<br>Yes: 1	0	
12	History of diabetic nephropathy	No: 0<br>Yes: 1	0	
13	History of diabetic neuropathy	No: 0<br>Yes: 1	0	
**Laboratory measures**				
14	HbA1c	< 8.9%: 0<br>≥ 8.9%: 1	0	Use the cut-off point of 75th percentile.
15	Albumin/Creatinin	<300: 0<br>≥300: 1	109 (0.7%)	
16	HDL cholesterol	≥ 0.95 mmol/l: 0<br>< 0.95 mmol/l: 1	147 (1.0%)	Use the cut-off point of 25th percentile
17	LDL cholesterol	< 2.92 mmol/l: 0<br>≥ 2.92 mmol/l: 1	151 (1.0%)	Use the cut-off point of 75th percentile
18	Total cholesterol	< 5.13 mmol/l: 0<br>≥ 5.13 mmol/l: 1	147 (1.0%)	Use the cut-off point of 75th percentile

19	Triglyceride	< 2.45 mmol/l: 0<br>≥ 2.45 mmol/l: 1	152 (1.0%)	Use the cut-off point of 75th percentile
20	Sodium (mmol/L)	135-145:0<br><135 or >145: 1	3	
21	Potassium (mmol/L)	3.5-6.0: 0<br><3.5 or >6.0: 1	9 (0.1%)	
22	Haemoglobin (g/L)	>110: 0<br>≤110: 1	157 (1.1%)	
**Other measures**				
23	Systolic blood pressure>=180	No: 0<br>Yes: 1	118 (0.8%)	
24	Diastolic blood pressure >=90	No: 0<br>Yes: 1	59 (0.4%)	
25	Underweight	No: 0<br>Yes: 1	23 (0.2%)	Recode from BMI <18.5
26	Diabetes duration (years)	< 19: 0<br>≥ 19: 1	11 (0.1%)	
27	Polypharmacy (≥5 drugs)	No: 0<br>Yes: 1	0	We calculate the total number of medications used at baseline and choose the cut point of 5

Supplementary Table 2. Incidence rates for outcomes in the study participants by frailty status

**Events**	**All (N=14543)**	**Frail (N=8080)**	**Non-frail (N=6463)**	**P**
Composite events (cardiovascular mortality/ myocardial infarction/ stroke)				
n/total (%)	1497/14543 (10.3%)	1021/8080 (12.6%)	476/6463 (7.4%)	<0.001
events/1000 patient-year	32.3	43.5	20.7	
All-cause mortality				
n/total (%)	1050/14543 (7.2%)	718/8080 (8.9%)	332/6463 (5.1%)	<0.001
events/1000 patient-year	21.4	28.9	13.7	
Cardiovascular mortality				

n/total (%)	703/14543 (4.8%)	517/8080 (6.4%)	186/6463 (2.9%)	<0.001
events/1000 patient-year	14.3	20.8	7.7	

Supplementary Table 3. Cox proportional hazards models of canagliflozin on composite events in frail and non-frail participants aged ≥ 65 (N=6621)

Hazard ratios (95% CI) for composite events (cardiovascular mortality, non-fatal myocardial infarction and non-fatal stroke) in participants **aged ≥65** (N=6621)	
Non-frail (FI≤0.25)	**0.77 (0.60–0.98)**
Frail (FI>0.25)	**0.82 (0.70–0.98)**
P for interaction between frailty and canagliflozin	0.615

Supplementary Table 4. Hazard ratios (95% CI) for safety outcomes in participants aged ≥ 65 (N=6621)

	Unadjusted HRs (95%CI)		
Outcomes	Frail (N=3718)	Non-frail (N=2903)	P for interaction between frailty and canagliflozin
Hypoglycemia	1.14 (0.91-1.41)	1.17 (0.84-1.62)	0.855
Urinary tract infection	0.96 (0.77-1.20)	1.30 (0.97-1.75)	0.088
Renal related adverse events	0.73 (0.59-0.90)	1.28 (0.80-2.03)	**0.031**
Fracture	1.05 (0.79-1.41)	1.18 (0.86-1.63)	0.521
Volume depletion	1.35 (1.02-1.78)	1.98 (1.27-3.09)	0.141
Osmotic diuresis	1.84 (1.18-2.86)	3.95 (2.38-6.55)	**0.027**
Hyperkalemia	0.88 (0.64-1.20)	0.80 (0.40-1.63)	0.741
Amputation	1.62 (1.04-2.53)	2.79 (0.93-8.36)	0.403
Acute kidney injury	0.62 (0.42-0.90)	0.85 (0.31-2.34)	0.581
Diabetic ketoacidosis	2.07 (0.40-10.68)	3.43 (0.40-29.57)	0.674
Female genital mycotic infection	9.31 (3.72-23.32)	8.18 (3.25-20.58)	0.832
Male genital mycotic infection	6.29 (3.00-13.19)	4.27 (2.66-6.87)	0.386

Supplementary Table 5. Cox proportional hazards models of canagliflozin on composite events by frailty level

	Hazard ratios (95% CI) for composite events (cardiovascular mortality, non-fatal myocardial infarction and non-fatal stroke)

Frailty level	All participants (N=14543)	Participants aged ≥65 (N=6621)
FI ≤0.25	0.90 (0.75 – 1.08)	**0.77 (0.60 – 0.98)**
FI 0.25-0.35	0.86 (0.73 – 1.02)	0.89 (0.71 – 1.13)
FI >0.35	**0.73 (0.61 – 0.88)**	**0.78 (0.61 – 0.99)**
P for interaction between frailty level and canagliflozin	0.107	0.932

Supplementary Table 6. Cox proportional hazards models of canagliflozin on safety outcomes by frailty level in all participants (N=14543)

Outcomes	Unadjusted HRs (95%CI) by frailty levels			
	FI ≤0.25 (N=6463)	FI 0.25-0.35 (N=5160)	FI >0.35 (N=2920)	P for interaction
Hypoglycemia	1.21 (0.99-1.49)	1.21 (0.99-1.47)	1.18 (0.92-1.51)	0.837
Urinary tract infection	1.45 (1.18-1.78)	1.08 (0.87-1.33)	0.98 (0.76-1.26)	**0.010**
Renal related adverse events	0.90 (0.67-1.23)	0.75 (0.61-0.91)	0.77 (0.64-0.93)	0.559
Fracture	1.41 (1.10-1.80)	1.19 (0.90-1.58)	0.99 (0.70-1.40)	0.096
Volume depletion	1.39 (1.03-1.87)	1.52 (1.15-2.00)	1.31 (0.96-1.78)	0.736
Osmotic diuresis	4.10 (2.87-5.86)	2.55 (1.70-3.82)	1.50 (0.91-2.45)	**<0.001**
Hyperkalemia	0.89 (0.55-1.44)	0.82 (0.61-1.10)	0.77 (0.57-1.04)	0.730
Amputation	1.97 (1.05-3.70)	1.74 (1.16-2.60)	1.23 (0.89-1.69)	0.119
Acute kidney injury	0.64 (0.35-1.17)	0.58 (0.38-0.89)	0.87 (0.62-1.22)	0.238
Diabetic ketoacidosis	1.90 (0.50-7.19)	2.99 (0.63-14.16)	57.45 (0.27-126.21)	0.096
Female genital mycotic infection	4.81 (2.96-7.80)	7.97 (4.01-15.85)	2.81 (1.32-5.98)	0.585
Male genital mycotic infection	3.92 (2.89-5.33)	4.67 (2.97-7.35)	14.48 (3.47-60.34)	0.095

Supplementary Table 7. Cox proportional hazards models of canagliflozin on composite events by frailty status in each trial separately

Frailty status	Hazard ratios (95% CI) for composite events (cardiovascular mortality, non-fatal myocardial infarction and non-fatal stroke)		
	CANVAS+CREDENCE participants (N=14543)	CANVAS participants only (N=10142)	CREDENCE participants only (N=4401)
Non-frail (FI≤0.25)	0.90 (0.75–1.08)	0.89 (0.74–1.08)	1.02 (0.53–1.94)
Frail (FI>0.25)	**0.79 (0.70–0.89)**	**0.82 (0.70–0.97)**	**0.77 (0.64–0.93)**
P for interaction between frailty and canagliflozin treatment	0.220	0.524	0.430

Supplementary Table 8. Cox proportional hazards models of canagliflozin on composite events by frailty level in each trial separately

Frailty level	Hazard ratios (95% CI) for composite events (cardiovascular mortality, non-fatal myocardial infarction and non-fatal stroke)		
	CANVAS+CREDENCE participants (N=14543)	CANVAS participants only (N=10142)	CREDENCE participants only (N=4401)
FI ≤0.25	0.90 (0.75 – 1.08)	0.89 (0.74 – 1.08)	1.02 (0.53 – 1.94)
FI 0.25-0.35	0.86 (0.73 – 1.02)	0.92 (0.75 – 1.13)	**0.73 (0.54 – 099)**
FI >0.35	**0.73 (0.61 – 0.88)**	**0.67 (0.50 – 0.89)**	**0.79 (0.63 –1.00)**
P for interaction between frailty level and canagliflozin	0.107	0.178	0.809
